# Large Language Model Versus Multidisciplinary Team: Feasibility Study of Pancreatic Cancer Management Recommendations

**DOI:** 10.2196/95411

**Published:** 2026-06-30

**Authors:** Zhuoran Liu, Xin Zhao, Tianyang Mao, Heng Zhou, Kangyi Jiang

**Affiliations:** 1 Department of Hepato-Pancreato-biliary Surgery, People’s Hospital of Leshan, No.238 Huian street, Shizhong District, Leshan, Sichuan Province, 614000, China, 86 833 211 9306; 2 Department of General Surgery, West China Hospital of Sichuan University, Chengdu, Sichuan, China

**Keywords:** pancreatic cancer, multidisciplinary team, large language models, clinical decision support, artificial intelligence, MDT, LLM

## Abstract

Of 269 pancreatic cancer multidisciplinary team cases, 125 complete structured cases were evaluated. The chat interface model labeled ChatGPT-5.2 matched the multidisciplinary team’s broad initial management category in 119 cases (95.2%, 95% CI 89.8%-98.2%). The end point was coarse, and the model was not version controlled, so findings only support supervised exploratory use.

## Introduction

Pancreatic cancer treatment decisions integrate resectability, metastatic disease, performance status, comorbidities, symptoms, and biomarkers [1,2]. Multidisciplinary team (MDT) review is recommended but may be limited by staffing and scheduling [3]. Large language models (LLMs) might help organize clinical information, yet their agreement with real pancreatic MDT decisions is uncertain [4]. We compared a chat interface LLM with documented MD recommendations as a feasibility assessment of prespecified broad management category concordance in complete structured cases, not autonomous decision-making or implementation.

## Methods

### Overview

We retrospectively reviewed deidentified pancreatic cancer cases discussed at weekly specialist pancreatic MDT meetings at a single center from March 2023 to December 2025. Of 269 MDT-discussed cases, 125 treatment-naive adults with pathologically confirmed pancreatic neoplasms and sufficient clinical, imaging, staging, and biomarker information for standardized prompts formed the analytic cohort. No purposive enrichment was used. The retained analytic file did not include a screening log, patient-level data for the 144 nonincluded cases, aggregate missing data descriptors, or exclusion categories. Thus, the cohort represents complete structured cases rather than all pancreatic cancer MDT cases, and optimistic selection bias is possible if excluded cases were more ambiguous, incomplete, or clinically complex. Two researchers extracted variables independently; a senior pancreatic cancer specialist adjudicated discrepancies.

The index LLM was the chat interface model labeled ChatGPT-5.2. This label reflects what was displayed during data collection, not an application programming interface–pinned public model identifier. Hidden model build, training data cutoff, temperature, system configuration, exact prompt entry dates, and replicate intersession outputs were not available or retained. No application programming interface–based or version-controlled access was used. All cases used the same structured prompt (Multimedia Appendix 1). The LLM workflow and study evaluation framework are shown in Multimedia Appendix 2. The primary outcome was exact case-level agreement between the LLM recommendation and MDT decision across 5 prespecified broad initial management categories. The categories were defined before concordance assessment and were not revised after reviewing model outputs. The end point did not evaluate regimen choice, sequencing, dose intensity, treatment line triggers, biomarker-specific regimen selection, or implementation safety. No rule-based or guideline-only comparator was constructed.

Three independent specialists rated each LLM output for comprehensiveness, perceived concordance, and rationality on 5-point Likert scales. Detailed mean specialist appraisal scores by specialty are shown in Multimedia Appendix 3. For perceived concordance, they saw the MDT decision; for other dimensions, they saw the case summary and LLM output. Because all dimensions were scored within the same workflow, halo effects and nonindependence are possible. Concordance proportions used Clopper-Pearson exact 95% CIs. Linearly weighted Cohen κ was used to assess rater consistency. Resectability, histology, and biomarker subgroup tabulations were descriptive only.

### Ethical Considerations

The Ethics Committee of Leshan People’s Hospital approved the study (LYLL2026KY117) and waived informed consent because retrospective deidentified data were used.

## Results

Among 125 included patients, the mean age was 62.36 (SD 8.92) years, 80 (64%) were male, 113 (90.4%) had pancreatic ductal adenocarcinoma, and 12 (9.6%) had grade 2 pancreatic neuroendocrine neoplasm (Table 1). The analytic cohort represented 46.5% of the 269 MDT-discussed cases. No data were available to compare included and nonincluded cases or to determine if exclusions reflected missing imaging, biomarkers, pathology, comorbidity details, or other contextual information. Overall broad category concordance was 119 (95.2%, 95% CI 89.8%‐98.2%; Table 2). Concordance was 32 of 36 (88.9%) in resectable disease, 38 of 38 (100%) in borderline resectable disease, 27 of 29 (93.1%) in locally advanced disease, and 22 of 22 (100%) in metastatic disease. These subgroup estimates are descriptive; 100% agreement in borderline resectable and metastatic disease should be interpreted with the strong constraint that stage and resectability impose on treatment direction. *BRCA1*/*2* mutant and microsatellite instability-high or mismatch repair-deficient (MSI-H/dMMR) rows are descriptive tabulations rather than powered subgroup analyses because the denominators were small.

Discordant cases mainly involved resectable or locally advanced disease and appeared related to different weighting of comorbidities, perioperative risk, and thresholds for neoadjuvant intensification. Mean specialist ratings were 3.21 (SD 0.58) for comprehensiveness, 3.85 (SD 0.42) for perceived concordance, and 3.97 (SD 0.39) for rationality. These ratings should not be interpreted as mutually independent dimensions or as validation of mechanical concordance because exposure to the MDT decision for one dimension could influence adjacent judgments. Weighted κ values ranged from 0.70 to 0.83, indicating substantial to near-perfect rater consistency only.

Concordance was defined as exact agreement between the LLM and MDT on the prespecified broad initial management strategy. Subgroup estimates, including biomarker subgroup estimates, are descriptive because no inferential subgroup analysis was prespecified, and some denominators were small.

**Table 1.** Baseline characteristics of included cases (N=125).

Characteristic	Value
Male sex, n (%)	80 (64.0)
Age (years), mean (SD)	62.36 (8.92)
BMI (kg/m^2^), mean (SD)	23.36 (3.27)
ECOG^a^ performance status 0, n (%)	93 (74.4)
ECOG performance status 1, n (%)	32 (25.6)
Diabetes mellitus, n (%)	63 (50.4)
Hypertension, n (%)	48 (38.4)
Cardiovascular disease, n (%)	18 (14.4)
CA19-9^b^ (U/mL), median (IQR)	897 (326‐3560)
Tumor in pancreatic head, n (%)	96 (76.8)
Resectable, n (%)	36 (28.8)
Borderline resectable, n (%)	38 (30.4)
Locally advanced, n (%)	29 (23.2)
Metastatic, n (%)	22 (17.6)
PDAC^c^, n (%)	113 (90.4)
pNET^d^ G2, n (%)	12 (9.6)
*BRCA1/2* mutant, n (%)	20 (16.0)
MSI-H/dMMR^e^, n (%)	4 (3.2)

a ECOG: Eastern Cooperative Oncology Group.

b CA19-9: carbohydrate antigen 19‐9.

c PDAC: pancreatic ductal adenocarcinoma.

d pNET G2: grade 2 pancreatic neuroendocrine neoplasm.

e MSI-H/dMMR: microsatellite instability-high or mismatch repair-deficient.

**Table 2.** Case-level concordance between the large language model and multidisciplinary team by subgroup.

Subgroup	Concordant/total, n	Concordance (%)	Exact 95% CI
Overall	119/125	95.2	89.8‐98.2
Resectable	32/36	88.9	73.9‐96.9
Borderline resectable	38/38	100.0	90.7‐100.0
Locally advanced	27/29	93.1	77.2‐99.2
Metastatic	22/22	100.0	84.6‐100.0
*BRCA1/2* mutant	20/20	100.0	83.2‐100.0
MSI-H/dMMR^a^	4/4	100.0	39.8‐100.0

a MSI-H/dMMR: microsatellite instability-high or mismatch repair-deficient.

## Discussion

In complete structured cases, the LLM often selected the same broad initial management category as the pancreatic MDT. This result should be interpreted narrowly. The 5-category end point captures treatment direction, not regimen-level correctness, safety, workflow benefit, patient benefit, or clinical equivalence. Because resectability and stage constrain initial management, especially in borderline resectable and metastatic disease, the 95.2% concordance is best viewed as the upper bound supported by this coarse end point, not evidence of nuanced multidisciplinary reasoning. Without a rule-based or guideline-only baseline, we cannot determine whether the LLM exceeded structured guideline retrieval or a simple decision tree [5]. More granular end points assessing regimen selection, sequencing, dose modification, biomarker-triggered treatment, radiation indications, operative risk trade-offs, or supportive care priorities would likely reduce observed concordance. The analysis mainly identifies feasibility issues, reporting constraints, and end point limitations for future studies.

The limitations are substantial. First, this was a single-center complete-case study; only 125 of 269 MDT-discussed cases were analyzed, and missing screening data prevented characterization of the 144 nonincluded cases. The estimate, therefore, applies only to complete structured cases and is likely optimistically biased. Second, standardized summaries may have simplified the MDT context by removing uncertainty, competing priorities, and workflow constraints. Third, the chat interface model was not reproducible because the model build, parameters, training data cutoff, prompt-entry dates, and replicate outputs were unavailable. Temporal validity is consequently uncertain for cases and guideline changes from March 2023 to December 2025. Fourth, most cases were pancreatic ductal adenocarcinoma, with only 12 grade 2 pancreatic neuroendocrine neoplasm cases; histology and biomarker subgroup results were descriptive, with small denominators and wide intervals, particularly for MSI-H/dMMR. Fifth, specialist ratings may be affected by halo effects because concordance scoring used the MDT decision. Finally, the MDT was an operational reference standard, not a gold standard. These findings are hypothesis-generating and support only cautious, supervised exploratory use, not autonomous clinical application.

## Supplementary material

10.2196/95411 Multimedia Appendix 1 Structured prompt template used for large language model evaluation.

10.2196/95411 Multimedia Appendix 2 Large language model workflow and study evaluation framework.

10.2196/95411 Multimedia Appendix 3 Mean specialist appraisal scores for comprehensiveness and perceived concordance with the multidisciplinary team decision.

## Abbreviations

LLM: large language model
MDT: multidisciplinary team
MSI-H/dMMR: microsatellite instability-high or mismatch repair-deficient
