# Supplementary material for: Large Language Model Versus Multidisciplinary Team: Feasibility Study of Pancreatic Cancer Management Recommendations
Source: J Med Internet Res. 2026 Jun 30;28:e95411. doi: 10.2196/95411 (PMC13318394; doi:10.2196/95411)
Supplement: Multimedia Appendix 1 [file jmir-v28-e95411-s001.docx]

Multimedia Appendix 1

**Structured Prompt Template Used for LLM Evaluation**

Structured prompt template used for all cases (with case-specific variables inserted into the bracketed fields):

You are assisting a specialist pancreatic cancer multidisciplinary team. Review the following de-identified case summary and provide an evidence-based recommendation for the initial management plan.

Case summary: [insert demographics, performance status, comorbidities, laboratory tests, imaging findings, pathology, genomic findings, and relevant symptoms].

Please respond in the following structure:

(1) clinical stage and resectability assessment;

(2) recommended initial management strategy;

(3) rationale from pancreatic surgery, medical oncology, and radiation oncology perspectives;

(4) biomarker-guided or supportive-care considerations if applicable; and

(5) additional information needed before implementation.

If the available information is insufficient, explicitly state the missing data and how it would affect the recommendation.

Note. The same structured prompt template was used for all included cases. The model was accessed through a chat-interface workflow using the displayed label ChatGPT-5.2. No API-pinned model identifier, fixed temperature setting, or parameter-controlled workflow was available. The study evaluation compared only broad initial management categories and did not assess regimen-level selection, treatment sequencing, dose intensity, or patient-level implementation safety.
