# Supplementary figures and images for: Large Language Model Versus Multidisciplinary Team: Feasibility Study of Pancreatic Cancer Management Recommendations
Source: J Med Internet Res. 2026 Jun 30;28:e95411. doi: 10.2196/95411 (PMC13318394; doi:10.2196/95411)

**Multimedia Appendix 2**


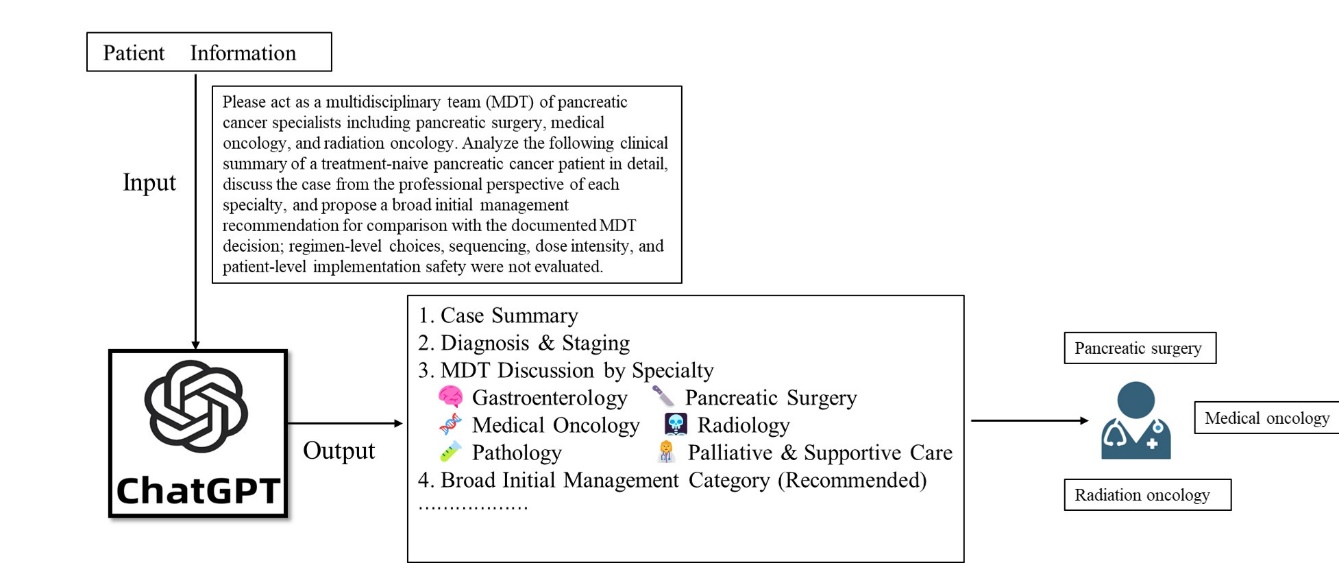


Figure S1. LLM workflow and study evaluation framework

Supplement: Multimedia Appendix 2 [file jmir-v28-e95411-s002.docx]
