# Supplementary material for: Large Language Model Versus Multidisciplinary Team: Feasibility Study of Pancreatic Cancer Management Recommendations
Source: J Med Internet Res. 2026 Jun 30;28:e95411. doi: 10.2196/95411 (PMC13318394; doi:10.2196/95411)
Supplement: Multimedia Appendix 3 [file jmir-v28-e95411-s003.docx]

**Multimedia Appendix 3**


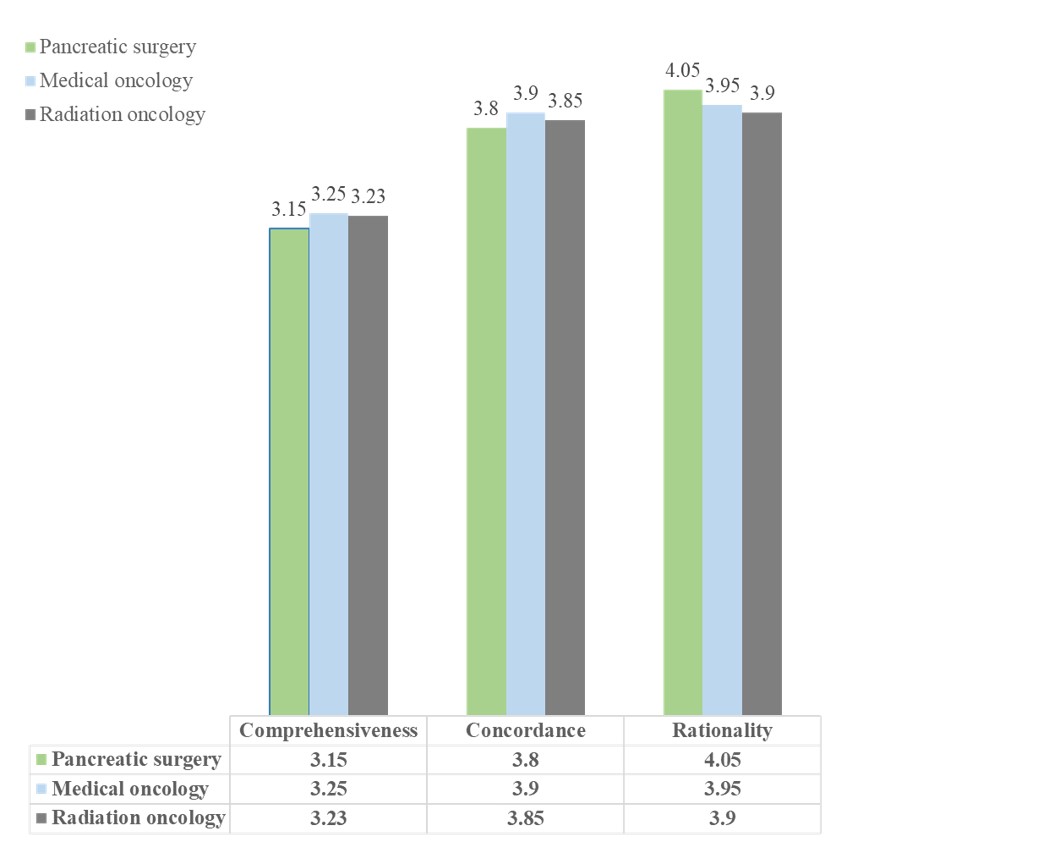


Figure S2. Mean specialist appraisal scores for comprehensiveness, perceived concordance with the MDT decision, and decision rationality of recommendations generated by the chat-interface model labeled ChatGPT-5.2. These descriptive ratings should not be interpreted as independent validation because the perceived-concordance dimension was scored with access to the MDT decision.
